# Heterologous Expression of Serine Hydroxymethyltransferase-3 From Rice Confers Tolerance to Salinity Stress in *E. coli* and Arabidopsis

**DOI:** 10.3389/fpls.2019.00217

**Published:** 2019-03-19

**Authors:** Pragya Mishra, Ajay Jain, Teruhiro Takabe, Yoshito Tanaka, Manisha Negi, Nisha Singh, Neha Jain, Vagish Mishra, R. Maniraj, S. L. Krishnamurthy, Rohini Sreevathsa, Nagendra K. Singh, Vandna Rai

**Affiliations:** ^1^ICAR-National Research Centre on Plant Biotechnology, New Delhi, India; ^2^Banasthali Vidyapith, Jaipur, India; ^3^Amity Institute of Biotechnology, Amity University, Jaipur, India; ^4^Plant Biotechnology Research Centre, Meijo University, Nagoya, Japan; ^5^ICAR Central Soil Salinity Research Institute, Karnal, India

**Keywords:** rice, Arabidopsis, salinity, serine, glycine, aquaporins

## Abstract

Among abiotic stresses, salt stress adversely affects growth and development in rice. Contrasting salt tolerant (CSR27), and salt sensitive (MI48) rice varieties provided information on an array of genes that may contribute for salt tolerance of rice. Earlier studies on transcriptome and proteome profiling led to the identification of salt stress-induced serine hydroxymethyltransferase-3 (*SHMT3*) gene. In the present study, the *SHMT3* gene was isolated from salt-tolerant (CSR27) rice. *OsSHMT3* exhibited salinity-stress induced accentuated and differential expression levels in different tissues of rice. *OsSHMT3* was overexpressed in *Escherichia coli* and assayed for enzymatic activity and modeling protein structure. Further, Arabidopsis transgenic plants overexpressing *OsSHMT3* exhibited tolerance toward salt stress. Comparative analyses of *OsSHMT3 vis a vis* wild type by ionomic, transcriptomic, and metabolic profiling, protein expression and analysis of various traits revealed a pivotal role of *OsSHMT3* in conferring tolerance toward salt stress. The gene can further be used in developing gene-based markers for salt stress to be employed in marker assisted breeding programs.

**HIGHLIGHTS:**

- The study provides information on mechanistic details of serine hydroxymethyl transferase gene for its salt tolerance in rice.

## Introduction

Rice (*Oryza sativa*) is a staple food for ∼90% of the Asian population (Mohanty et al., 2013). Production of rice in 2011–2014 was 495.63 million tons (MT) (FAO, 2014 ^1^). The global population is projected to increase by 25% by 2050 (United Nations World Population Prospects, 2017 ^2^). Therefore, there is a need for a commensurate increase in rice production to meet the ever-increasing demands of the growing population (FAOSTAT, 2009 ^3^). Although India has the largest acreage under rice [44.5 million ha (Mha)], the average productivity (2.5 tons ha^-1^) is abysmally low compared with China (Singh et al., 2016). This low productivity could be attributed to harsh conditions in different agroclimatic regions of India under which rice is cultivated and often subjected to various abiotic stresses (salinity, drought, flood, and nutrient deficiencies).

In India, a large proportion of agriculture land (6.73 Mha) is affected by high saline content (Singh et al., 2016). Salinity adversely affects growth and development, particularly during seedling and flowering stages, and consequently the yield potential of rice (Krishnamurthy et al., 2016; Zeng et al., 2018; Morton et al., 2019). The salinity-tolerant plants have evolved an array of adaptive strategies (exclusion, compartmentation, and secretion of Na^+^) to mitigate the toxic effects of salinity stress (Arzani and Ashraf, 2016; Zeng et al., 2018). Potassium (K) and calcium (Ca) signaling also exert significant influences toward conferring tolerance toward salinity stress in rice (Shabala and Cuin, 2008; Frouin et al., 2018).

Reactive oxygen species (ROS) and osmolytes (choline, glycine betaine, sugars, etc.) have also been implicated in conferring variable levels of tolerance toward salt stress (Hanson et al., 2000). Complete sequencing of rice genome (IRGSP, 2005) has expedited the process of deciphering the molecular entities, which play pivotal roles in conferring tolerance to salt stress. Availability of salt-tolerant and salt-sensitive rice varieties and state-of-the-art omics technologies further provided the fillip in this endeavor.

Serine hydroxymethyltransferase (SHMT), a pyridoxal phosphate-dependent enzyme, plays a pivotal role in cellular one-carbon pathways by catalyzing the reversible conversions of L-serine to glycine and tetrahydrofolate to 5,10-methylenetetrahydrofolate in lower and higher organisms (Schirch and Szebenyi, 2005). Structurally conserved SHMT has been implicated in various roles across lower and higher organisms (Moreno et al., 2005; Voll et al., 2006). In plants, SHMT activity has been detected in different organelles (mitochondria, cytosol, plastids, and nuclei) suggesting their diverse roles in metabolic pathways (Besson et al., 1995; Neuburger et al., 1996; Zhang et al., 2010). In rice, the *SHMT* family comprises five members (*OsSHMT1-5*). *OsSHMT1*, encoding the largest subunit of SHMT and an ortholog of *Arabidopsis thaliana SHM1*, was identified by employing enhancer trapping and the characterization of chlorophyll-deficient mutant (*oscdm1*) (Wu et al., 2015), photorespiratory mutant *osshm1* and map-based cloning (Wang et al., 2015).

In Arabidopsis also, the mutation in *AtSHM1* (At4g37930) caused an aberration in mitochondrial SHMT activity and exhibited a lethal photorespiratory phenotype during growth at ambient CO_2_ (Somerville and Ogren, 1981; Voll et al., 2006). These studies suggested conserved function of SHMT1 in photorespiration in taxonomically diverse rice and Arabidopsis. Interestingly, in soybean, SHMT plays a pivotal role in cyst nematode (SCN) resistance (Liu et al., 2012; Kandoth et al., 2017). In another study, overexpression of *ApSHMT* from halotolerant cyanobacteria *Aphanothece halophytica* in *Escherichia coli* (*E. coli*) triggered a higher accumulation of glycine betaine due to elevated levels of precursors, choline and serine, and consequently augmented tolerance toward salinity-stress (Waditee-Sirisattha et al., 2012). These studies thus suggested roles of SHMT not only in photorespiration but also in other metabolic pathways in different plant species.

The recombinant inbred line (RIL) population of salt-tolerant (CSR27) and salt-sensitive (MI48) rice genotypes were screened for their extreme tolerance and sensitivity toward salt stress, and bulk segregant analysis together with a gene expression study led to the identification of an array of differentially regulated salt stress responsive genes including *OsSHMT3* (Pandit et al., 2010) using bulk tolerant (BT) and bulk sensitive (BS) RILs. In a subsequent study, these BT and BS populations were also analyzed for their proteomic profiling, which revealed high expression of several proteins including serine hydroxymethyltransferase-3 (OsSHMT3) in the former (Mishra et al., 2016). Overexpression of salt-tolerance-related genes as well as stress-inducible transcription factors has led to the transgenic plants with enhanced salt tolerance (Arzani and Ashraf, 2016). Overexpression of OsCYP94C2, (gene from Cyt450 family) and C-terminal centrin-like domain (OsCCD1) conferred tolerance toward salt stress (Kurotani et al., 2015; Jing et al., 2016). In this context, it is intriguing whether overexpression of *OsSHMT3* would elicit any tolerance toward salt stress.

Here, we examined the role of SHMT in conferring salt stress tolerance by mediating biosynthetic pathway of glycine to serine interconversion and synthesis of amino acids. In the present study the *OsSHMT3* was amplified from salt-tolerant rice. *OsSHMT3* was overexpressed in *E. coli* and assayed for enzymatic activity and modeling protein structure. Further, transgenic Arabidopsis overexpressing *OsSHMT3* (*OEs*) lines were tested for their tolerance toward salt stress. Comprehensive and comparative analyses of the wild-types and *OEs* for their ionomic, transcriptomic and metabolic profiles, protein expression and different traits were employed to study the role of *OsSHMT3* in conferring tolerance toward salt stress in Arabidopsis.

## Materials and Methods

### Materials and Growth Conditions

Salt-tolerant (CSR11, CSR27, CSR36, and K478) and salt-sensitive (MI48, IR29, and K198) rice (*Oryza sativa* ssp. *indica*) lines were used in the present study. K478 and K198 are the wild introgression lines derived from the cross of *O. sativa* and *O. rufipogon* (Pushpalatha et al., 2016). Seeds of all the rice lines were surface-sterilized with 1% (w/v) NaClO, washed with distilled water (3 to 4 times), soaked overnight in deionized (Milli-Q) water, and then were sown on the germination paper. After 3 days, germinated seeds were transferred to the hydroponic system comprising standard polycarbonate transparent Magenta (GA-7) box (width × length × height = 75 mm × 74 mm × 138 mm) as described (Negi et al., 2016), containing 200 ml of one-half-strength Hoagland’s nutrient solution. The hydroponic system was maintained under controlled growth conditions (16-h day/8-h night cycle at 24 ± 2°C, relative humidity of ∼70–80%, and photosynthetic photon flux density of 250–350 μmol m^-2^ s^-1^ provided by the florescent tubes). The hydroponic system was aerated by using an aquarium air pump (power 5 W and pressure 2 × 0.02 MPa). After germination, seedlings (14-day-old) were subjected to 150 mM NaCl stress for 24 h or as required for the experiment. *A. thaliana* (Col-0) seeds were used for the generation of the transgenics. *E. coli* (BL21) cells were grown in Luria Broth (LB) medium supplemented with different concentrations of NaCl (100–700 mM) for 12 h. All the experiments were carried out in three biological replicates.

### RNA Extraction and Gene Expression Analysis

Total RNA was extracted from the shoot and root tissues of the salt-tolerant and salt-sensitive rice seedlings after subjecting them to NaCl stress for different time intervals (0–24 h) by using RNeasy kit (Qiagen). Trace amount of DNA was removed by RNase-free DNase treatment. The RNA quality was analyzed on 1.2% (w/v) formaldehyde gel and quantified with NanoDrop 1000 Spectrophotometer (Thermo Scientific, United States). The primers for the Real-time PCR were designed from the exonic sequences using IDT SciTools software. First-strand cDNA was synthesized using oligo (dT)-18 primer and reverse transcribed using Superscript II^TM^ Reverse Transcriptase (Invitrogen). Eukaryotic *elongation factor1^TM^-alpha* (*EF1A*) was used as an internal control. The RT-qPCR analysis was performed by using SuperScript III Platinum SYBR Green One-Step RT-qPCR Kit (Invitrogen) in a Stratagene Mx3000P qPCR system (Agilent Technologies, United States). The data were analyzed by Mxpro-QPCR software (Stratagene). Relative expression levels of the genes were computed by the 2^-ΔΔ*C*_T_^ method of relative quantification (Livak and Schmittgen, 2001). All the gene-specific primers used are listed in Supplementary Table S1. The RT-qPCR was done using six biological replicates.

### Search for SHMT Homologs

Serine hydroxymethyltransferase homologs were searched in the genomes of *A. halophytica* (Ap), *E. coli* (Ec), *Neurospora crassa* (Nc), *Saccharomyces cerevisiae* (Sc), *A. thaliana* (At), *O. sativa* (Os), *Zea mays* (Zm), *Glycine max* (Gm), *Pisum sativum* (Ps), *Triticum aestivum (Ta)*, *Solanum tuberosum* (St), and *Homo sapiens* (Hs) by employing BLASTP or tBLASTn against TAIR^4^, TIGR^5^, NCBI^6^, and unigene. The protein sequences were confirmed using Pfam database^7^. Multiple sequence alignment and phylogenetic analysis by the neighbor-joining method were carried out by employing Clustal Omega (Version 1.2.0) and FigTree (Version 1.4.0), respectively.

### Cloning and Overexpression of *OsSHMT3* in *E. coli* and *Arabidopsis*

The *OsSHMT3* coding sequence was amplified from the cDNA of salt-stressed (150 mM NaCl) CSR27 shoot, using forward and reverse primers with *Eco*R1 and *Xho*1 restriction sites, and cloned into pET29a expression vector with N-terminal 6 × His tag. The construct was sequenced to confirm the correct orientation of *OsSHMT3*. The construct was then transformed into *E. coli* strains BL21 (DE3) and DH5α. For transformation in Arabidopsis, the forward and reverse primers were designed with *Xba*1 and *Sa*l1 restriction sites, respectively. The pORE-E2 expression vector with the pENTCUP2 promoter (Coutu et al., 2007) was used for transformation into Arabidopsis. CSR27 seedlings were subjected to NaCl stress (150 mM) for 24 h, and the shoot was harvested for the extraction of mRNA and subsequent synthesis of cDNA. *OsSHMT3* was amplified using gradient PCR (50 to 60°C). PCR product was purified from the gel and double digested with *Xba*1 and *Sal*1, then ligated to *Xba*1 and *Sal*1 digested binary pORE-E2 vector. After ligation, the construct was mobilized to *E. coli* and Agrobacterium strain LBA440. Recombinants were confirmed by colony PCR and sequencing was carried out to confirm the validity of *OsSHMT3*. The sequence was submitted to NCBI (accession number MG799132). Agrobacterium transformed with *OsSHMT3* was then used for generating transgenic lines of Arabidopsis using the floral dip transformation method (Clough and Bent, 1998). Positive transformants were selected on kanamycin (50 μg/ml) plates and verified by PCR using *OsSHMT3*-specific primers. T3 generation was selected for further studies (Supplementary Figure S4). The primers used are listed in Supplementary Table S2.

### Recombinant Protein Purification

*Escherichia coli* BL21 (DE3) was transformed with the recombinant plasmid. A single colony was inoculated into LB medium containing antibiotic (Kanamycin, 50 μg mL^-1^) and the culture was incubated at 200 rpm (37°C). Culture was induced with 1 mM IPTG at 0.6–0.8 OD. SDS–PAGE was used for checking the expression. Cell pellets were sonicated in a solution containing 50 mM Tris (pH 7.4) and 300 mM NaCl. The protein found in the inclusion bodies was extracted with a solution containing 50 mM Tris (pH 7.4), 300 mM NaCl and 6M urea. The extract was centrifuged and supernatant was loaded onto Ni-NTA column for purification. Eluted fractions were analyzed on 10% SDS–PAGE. The fractions were pooled and gradient dialyzed against 1X PBS with varying concentrations of urea for refolding. The final protein was re-suspended in 1X PBS. Protein was analyzed on SDS–PAGE and quantified using Bradford assay.

### Protein Modeling

Template search with Blast and HHblits was performed against the SWISS-MODEL Template Library (SMTL). The SHMT sequence searched with BLAST (Altschul et al., 1997) against the primary amino acid sequence present in SMTL resulted in the identification of 47 templates. An initial HHblits profile was built as described (Remmert et al., 2011), followed by 1 iteration of HHblits against NR20. The obtained profile was then searched against all the profiles of SMTL, which resulted in the identification of 526 templates. For each identified template, its quality was predicted from the features of the target-template alignment. The templates with the highest quality were then selected for building the model based on the target-template alignment using ProModII. Models are built coordinates, which are conserved between the target and the template and copied from the template to the model. Insertions and deletions were remodeled using a fragment library. Side chains were then rebuilt. Finally, the geometry of the resulting model was regularized using a force field. When loop modeling with ProModII (Guex and Peitsch, 1997) did not give satisfactory results, an alternative model was built with MODELLER (Sali and Blundell, 1993). The global and per-residue model quality was assessed using the QMEAN scoring function (Benkert et al., 2011). For improved performance, weights of the individual QMEAN terms were trained specifically for SWISS-MODEL.

### OsSHMT Activity

Recombinant proteins purified through Ni-NTA column were used for the enzymatic assays. The SHMT (EC 2.1.2.1) activity was assayed as described (Simic et al., 2002) in three biological replicates. The reaction mixture comprised Tris–Hcl buffer (50 mM, pH 9.0), L-serine (200 mM), 5,10-methylenetetrahydrofolate (18 mM), Pyridoxal-L-phosphate (2 mM), and 100 μL enzyme in a final volume of 1.0 mL. The reaction mixture was incubated at 25°C for 15 min and 125 μL of 25% trichloroacetic acid (w/v) was added to 500 μL of sample. The reaction mixture was centrifuged at 4°C and the supernatant was neutralized with the buffer (31.8 g of K_2_CO_3_ in 100 mL of 20 mM Tris–Hcl, pH 8.0). Glycine was analyzed by an amino acid analyzer with a Shim-pack Li column (Shimadzu, Kyoto, Japan).

### Amino Acids Analysis

OsSHMT3 overexpressing and control *E. coli* cells in triplicate were homogenized in absolute methanol and centrifuged. The supernatant was assembled and the pellet was extracted again with 90% (v/v) methanol. The pooled methanol extract was dried at 45°C in a vacuum rotary evaporator. Dried samples were dissolved in mobile-phase (MA) solution pH 2.6, 14.1 g of trilithium citrate tetrahydrate, 70 mL of 2-methoxyethanol, and 13.3 mL of 60% HClO_4_, and injected into an amino acid analyzer (Shimadzu, Kyoto, Japan) for separation. Amino acid was extracted from Arabidopsis leaves as described (Waditee et al., 2005).

### Metabolite Quantification

Glycine betaine (GB) and choline were extracted from the control and *E. coli* cells expressing *OsSHMT*. *E. coli* cells were grown in liquid LB media containing various concentrations of NaCl (0, 100, 300, and 500 mM) in three replicates. Log phase cultures were taken for GB and choline extraction using KI-I_2_ method as described (Hibino et al., 2002). GB and choline were then quantified on a Time-of-flight mass spectrometer (AXIMA CFR, Shimadzu/Kratos, Japan) with d^9^-choline and d^11^-betaine, respectively, as an internal standard.

### SDS–PAGE and Western Blot Analyses

SDS–PAGE and Western blot analyses were performed according to the standard protocols. Anti- OsSHMT was developed through injection of the purified recombinant protein into a goat (GeNei, Banglore, India). Bradford method was used for assaying the protein quantity. Coomassie brilliant blue (CBB) dye was employed for observing the protein bands on SDS–PAGE.

### Assessment of Salt Stress Tolerance in Transgenic Arabidopsis

Seeds were surface-sterilized with 1% (w/v) NaClO and 0.05% (v/v) Tween-20 for 8 min and washed 4–5 times with sterile water. Surface-sterilized seeds were sown on a Petri plate made with one-half strength Murashige and Skoog (MS) medium, 0.8% (w/v) agar, and 3% (w/v) sucrose. After stratification for 2d at 4°C in the dark, plates were transferred to controlled condition in a Plant Growth Chamber (MLR-351, Sanyo, Japan) set at 16 h/8 h light dark cycle and a light intensity of ∼100 μmol photons m^-2^ s^-1^. Salt stress tolerance was analyzed using two homozygous transgenic lines (OE3 and OE5) overexpressing *OsSHMT*3 in T3 generation of Arabidopsis. Seedlings of the wild-type and transgenics after 7 d of germination were transferred to MS agar plate (90 × 18 mm) supplemented with different concentrations of NaCl (0, 100, 150, and 200 mM). After 10 days growth, seedlings of the wild-type and transgenics were harvested and analyzed for their morphophysiological responses. ImageJ software was used for measuring the root length from the scanned images. For documenting the physiological data, 7-day-old seedlings after germination on MS agar plates were transferred to vermiculite culture in pots (70 mm diameter) with one-half strength MS solution supplemented with different concentrations of NaCl (0, 100, 150, and 200 mM) in three replicates with 3 plants in each pot.

### Quantification of Chlorophyll, Anthocyanin Content and Photosynthetic Activity

Chlorophyll content was quantified as described (Palta, 1990). Control and salt stress leaves of three replicates of Arabidopsis were harvested, their fresh weight recorded, homogenized with 80% (v/v) acetone, and incubated overnight at -20°C. Samples were centrifuged (13,200 × *g*) for 10–15 min, and supernatant was used for absorbance at 663 and 645. The amount of total chlorophyll was calculated as: 20.2× the absorbance at 645 + 8.02× the absorbance at 663 nm. Anthocyanin was isolated from the shoot of Arabidopsis as described (Lange et al., 1971), and the absorbance was recorded at 532 and 653 nm. The overlap of chlorophyll was nullified by subtracting 0.24 from the absorbance value at 653 nm (Murray and Hackett, 1991). A molar extinction coefficient (e) with corrected absorbance was used for the quantification as described (Hrazdina et al., 1982). Final values were computed for 1 g of fresh weight (FW) and denoted in mg/g FW units. Photosystem II (PS II) activity was analyzed by a Mini PAM fluorometer (PAM-2000, Walz, Germany). At 25°C, the leaves were kept in dark for 30 min, and data was acquired with software (DA-2000, Walz) as described (Hoshida et al., 2000).

### Assays of Antioxidant Enzymes Superoxide Dismutase (SOD) and Peroxide Dismutase (POD)

Wild-type, OE3 and OE5 seedlings (14-day-old) were grown on MS agar medium, and then transferred to MS agar medium supplemented with 100 mM NaCl for one week. Enzymes were extracted from the leaf tissues for assaying the activity of POD (EC 1.11.1.7) and SOD (EC 1.15.1.1). Leaf tissues were homogenized in 2 ml each of potassium phosphate buffer (150 mM, pH 6.1) for POD, and phosphate buffer (pH 7.8) with EDTA (3 mM) for SOD. After centrifugation (12,000 × *g*) for 20 min at 4°C, the supernatant was used for assaying the activities of SOD and POD. POD was analyzed as described (Zhang, 1992). Briefly, the reaction mixture comprised potassium phosphate buffer (50 mM, pH 6.1), 1% guaiacol (w/v), 0.4% H_2_O_2_ (v/v), and the 50 μL of enzyme extract. The absorbance was recorded at 470 nm. SOD was quantified as described (Giannopolitis and Ries, 1977). The reaction mixture comprised riboflavin (1.3 μM), methionine (13 mM), NBT (63 μM), sodium carbonate (0.05 M, pH 10.2), and 50 μL of enzyme extract. The absorbance was recorded at 560 nm. The activities of SOD and POD were represented as unit mg protein^-1^. The Lowry et al. (1951) method was used for protein quantification as described. All the observations were taken in three replicates.

### Ion Quantification

Na^+^ and K^+^ ions were quantified in root, stem and leaf tissues as described (Waditee et al., 2007). Briefly, the root, stem and leaf from the control and NaCl-stressed seedlings of three replicates were homogenized in sterile distilled water (1 ml) and analyzed using Shimadzu PIA-1000 Personal Ion Analyzer (Shimadzu, Japan).

### Microarray Analysis

Microarray analysis was carried out to determine the effect of salt stress on a global genome expression profiling in the replicated samples of wild-type and transgenic line (OE3) of *Arabidopsis* seedlings by microarray technique. The control and NaCl (150 mM)-stressed (24 h) seedlings (14-day-old) for 24 h of wild-type and OE5 were used for the extraction of total RNA in triplicate using RNeasy kit (Qiagen). RNA were then labeled with AffymetrixGeneChip^®^ 3′ IVT Express Kit. Affymetrix^®^ gene chips (ATH1) of Arabidopsis were hybridized and washed with Affymetrix^®^ GeneChip^®^ Fluidics Station 450 and scanned by GeneChip Scanner 3000. The scanned images were then analyzed with Affymetrix GeneChip^TM^ Command Console Software (AGCC). R-package was used to analyze the CEL files generated from the AGCC. *P*-values for the differentially expressed genes were set at <0.05 and log fold-change >2. The data were submitted to Gene Expression Omnibus (accession number GSE109283).

### Statistics

The data were subjected to two-way ANOVA, and the significant differences for groups of data were computed with Fisher’s LSD (with *P* < 0.05) using Statistical Analysis Software (SAS, version 9.2).

## Results

### Effect of Salt Stress on *OsSHMT3* Expression

**FIGURE 1:**
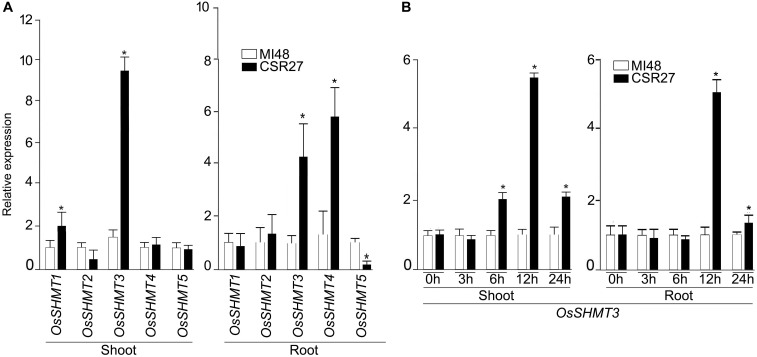
Effect of salt stress on the relative expression levels of *OsSHMTs* in contrasting sensitive and tolerant rice cultivars. Seedlings (14-day-old) of MI48 and CSR27 were subjected to 150 mM NaCl stress for different time intervals. Real-time PCR analyses were carried out for determining the relative expression levels in the shoot and root of **(A)** *OsSHMT 1–5* after 24 h and **(B)** *OsSHMT3* at different time points. Data (*n* = 9) represented values that were generated from three independent biological replicates with 3 technical replicate each. Significant difference (one-way ANOVA) between MI48 and CSR27 is indicated with an asterisk (*P* < 0.05).

In rice, *OsSHMT* family is represented by 5 members hereafter referred as *OsSHMT1-5*. Relative expression levels of *OsSHMT2*, *4*, and *5* in shoot and *1, 2* in roots were comparable in MI48 and CSR27. The expression levels of *SHMT1* and *SHMT3* were significantly higher in shoot tissues of CSR27 as compared with MI48, whereas, mRNA levels of *SHMT3* and *SHMT4* were significantly increased in roots of CSR27 as compared with MI48 (Figure 1A). Since *OsSHMT3* showed higher expression in both root and shoot tissues, it was further employed to determine genotypic-specific response or more of a generic manifestation of salinity-tolerant genotypes. Therefore, the relative expression levels of *SHMT3* were assayed in shoots and root of representative members of salt-sensitive (K198, IR29) and salt-tolerant (CSR11, CSR36, and K478) genotypes (Supplementary Figure S1). Further, to determine whether salt stress exerts influence on *OsSHMT3* temporally, its expression levels were evaluated in shoot and roots of MI48 and CSR27 that had been subjected to salt stress for different time intervals (Figure 1B). After 12 h of salt stress treatment, the expression of *SHMT3* was found to be optimal in both shoot and root of CSR27 compared with MI48.

### Sequence Analysis

It was evident from the phylogenic tree (Supplementary Figure S2) that SHMT protein sequences could be categorized into three sub groups as I, II, and III. Group I was further divided into 3 subgroups: subgroup IA comprised of ApSHMT and EcSHMT; subgroup IB had a cluster of AtSHMT5, TaSHMT, OsSHMT4, OsSHMT5, ZmSHMT2, AtSHMT4, and GmSHMT; subgroup IC comprised of OsSHMT2, OsSHMT1, ZmSHMT1, AtSHMT6, AtSHMT7, and HsSHMT2. Group II had an exceptionally member, AtSHMT3 along with SHMT1 and SHMT2 from human, OsSHMT3 was seen to belong to Group IIIA. It was observed that rice, *P. sativum* and *S. tuberosum*, had one gene *OsSHMT*3, *PsSHMT, StSHMT*, respectively, that belonged to this group whereas Arabidopsis had two genes (*AtSHMT1 and AtSHMT2*).

### Enzymatic and Salt Tolerance Properties of OsSHMT

**FIGURE 2:**
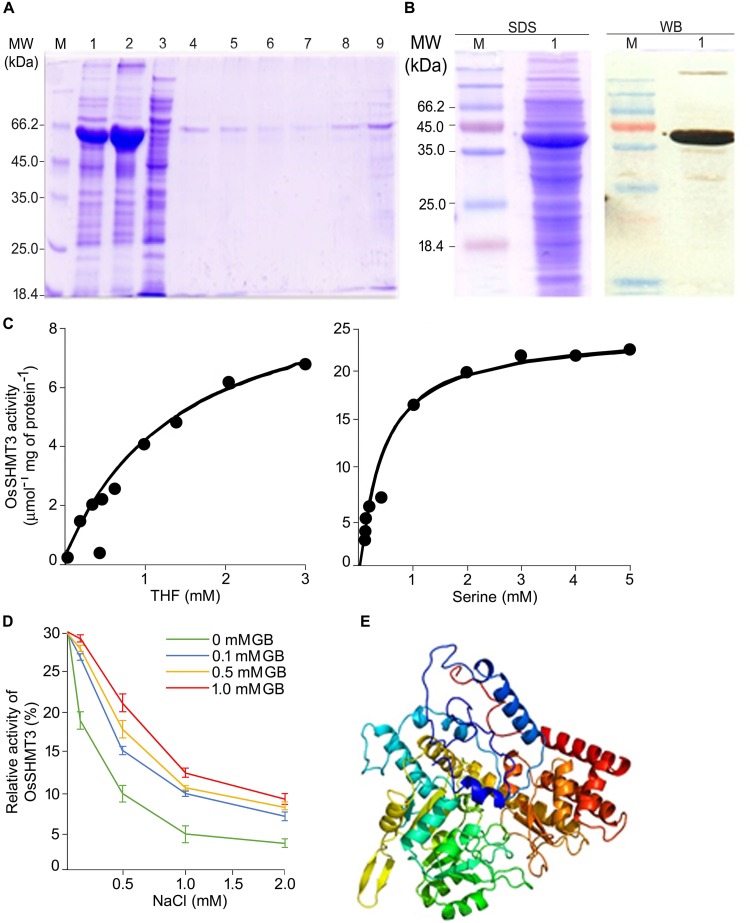
Properties of OsSHMT enzyme. **(A)** Purification of recombinant OsSHMT. Eluted fractions from Ni-NTA column were analyzed on 10% (w/v) SDS–PAGE. Lanes M: marker; 1: total protein of induced sample; 2: sonicated pellet; 3: sonicated supernatant; 4: flow through; 5–9: elution 1–5 **(B)** Purified protein (left panel) and its Western blot analysis (right panel) showing the expression of OsSHMT3 using anti-6-His (6X-His tag) secondary antibody **(C)** Enzyme activity of OsSHMT3 in the presence of tetrahydrofolate (THF) and L-serine **(D)** THF-dependent per cent relative enzymatic activity of the purified OsSHMT3 was assayed in the presence of different concentrations of NaCl and glycine betaine (GB). The enzyme activity measured at 0 mM NaCl was taken as 100%. **(E)** 3D structure of OsSHMT3 protein. The modeling was done using modeler software with X-ray crystallographically resolved homologous structure available on PDB database.

The OsSHMT3 gene was amplified from salt-tolerant rice and cloned in protein expression vector pET29a. OsSHMT3 protein formed inclusion bodies thus, extracted and purified by Ni-NTA column (Figure 2A) to assess quantity of protein. The Ni-NTA column elute from 7 to 9 were pooled and dialyzed with urea for refolding (Colangeli et al., 1998) to immobilized divalent metal to get active protein for study of enzyme kinetics (Figure 2B). OsSHMT3 activity was detected in the presence of L-serine and THF as substrate (Figure 2C). Serine concentration varied from 0 to 5 mM and THF was kept constant at 0.9 mM. When THF was used as substrate THF concentration varied from 0 to 3 mM. The kinetic parameter was derived from non-linear regression data fitting (GraphPad Prism 6). The *K_m_* and *V_max_* values (data not shown) for both serine and THF followed Michaelis-Menten kinetics.

Salt sensitivity of OsSHMT3 was observed as the enzyme activity decreased (30–90%) with increasing levels of (100–2000 mM) NaCl (Figure 2D). Since glycine betaine (GB), a known osmolyte, was used to observe protection of SHMT from salt stress, 100 to 1000 mM of GB was supplemented to NaCl for detecting enzyme activity. Interestingly 100 mM GB could recuperate SHMT activity from 40 to 96% at 100–1000 mM NaCl. Glycine betaine could not only protect efficiently but also helped in enhancing activity as it was 1.6-fold higher in 2 M NaCl when 1 mM GB was added.

**FIGURE 3:**
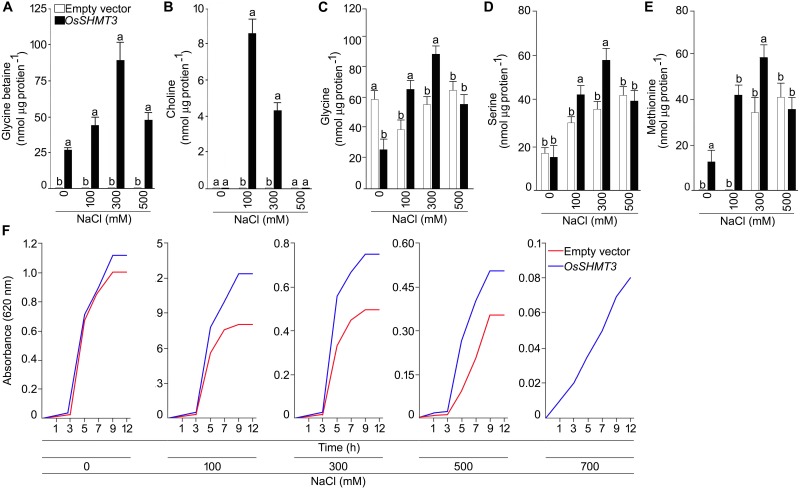
Analysis of *OsSHMT3* overexpressing *Escherichia coli* for salt stress tolerance. *E. coli* (BL21) cells grown in Luria Broth (LB) medium were subjected to salinity stress (100–500 mM NaCl) and assayed for the concentrations of **(A)** glycine betaine, **(B)** choline, **(C)** glycine, **(D)** serine, and **(E)** methionine were quantified. **(F)** *E. coli* (BL21) cells were grown in LB medium and the effect of different concentrations of NaCl (100–700 mM NaCl) on their temporal growth profile was assayed at absorbance 620 nm. Values (*n* = 9) are mean ± SE and different letters on the histograms indicate that the values differ significantly (one-way ANOVA; *P* < 0.05).

To gain insight into the structural details of SHMT, a model was obtained by applying homology modeling. The 62% structural homology is with mitochondrial and 58% with cytosolic SHMT. It was observed that the protein is a homo-tetramer, as are other members of SHMT. The structural motif was preserved as the sequence being conserved with 60% identical template. A dependable 3D-model based on human and Arabidopsis, could be constructed for OsSHMT3. The residues contributing for OsSHMT3 catalysis for binding of PLP and pteridine were conserved. Residues for aldimine formation and iron paring with PLP (Lys286 and Asp257), and for external aldimine interaction (Tyr112, His260, and Arg430), and with 5′-phosphate of PLP (Tyr102 and His285), and with folate (Asn415 and Tyr111), are all conserved among AtSHMT1, 2 and OsSHTM3 (Figure 2E). The OsSHMT3 was stable due to rigid entry sites of tetramer as reported for PDB based X ray resolved structural model of Arabidopsis protein. SHMT is a key enzyme for the serine biosynthetic pathway, hence, it is pertinent to analyze glycine, serine and methionine amino acids in *OsSHMT3* overexpressed *E. coli* and plants expressing *OsSHMT3*. Here, *E. coli* cells (BL21) expressing *OsSHMT3* and an empty vector were subjected to 0–500 mM NaCl stress and were investigated for free amino acids (Figure 3C–E). GB and choline were detected in *OsSHMT3* overexpressing *E. coli* after salt stress. The GB content was enhanced during salt stress condition and highest (two fold) at 300 mM NaCl (Figure 3A). Similarly, choline was high at higher salinity level and it reached maximum at 100 mM NaCl (8.5-fold) (Figure 3B).

Glycine, serine, and methionine free amino acids were found to be augmented at higher levels of salinity (Figure 3C–E). Glycine was higher (50%) in *OsSHMT3* overexpressing cells at 100 and 300 mM NaCl in comparison with control cells (empty vector) (Figure 3C). Likewise, serine was also showing a similar trend and increased up to 300 mM NaCl (Figure 3D). At 500 mM NaCl glycine and serine both showed inhibition. Methionine amino acid increased until 300 mM NaCl was reached in *OsSHMT3* overexpressing cells. However, in control cells methionine could not be detected at 0 and 100 mM NaCl, and at 300 and 500 mM NaCl its levels were lower in comparison with *OsSHMT3* (Figure 3E). The growth of *OsSHMT3* overexpressing *E. coli* was always higher than that of control *E. coli* cells (Figure 3F). At 700 mM NaCl control cells could not grow, however, *OsSHMT3* could grow and hence demonstrated its salt tolerance ability.

### Overexpression of OsSHMT3 Enhances Salt-Tolerance in Arabidopsis

**FIGURE 4:**
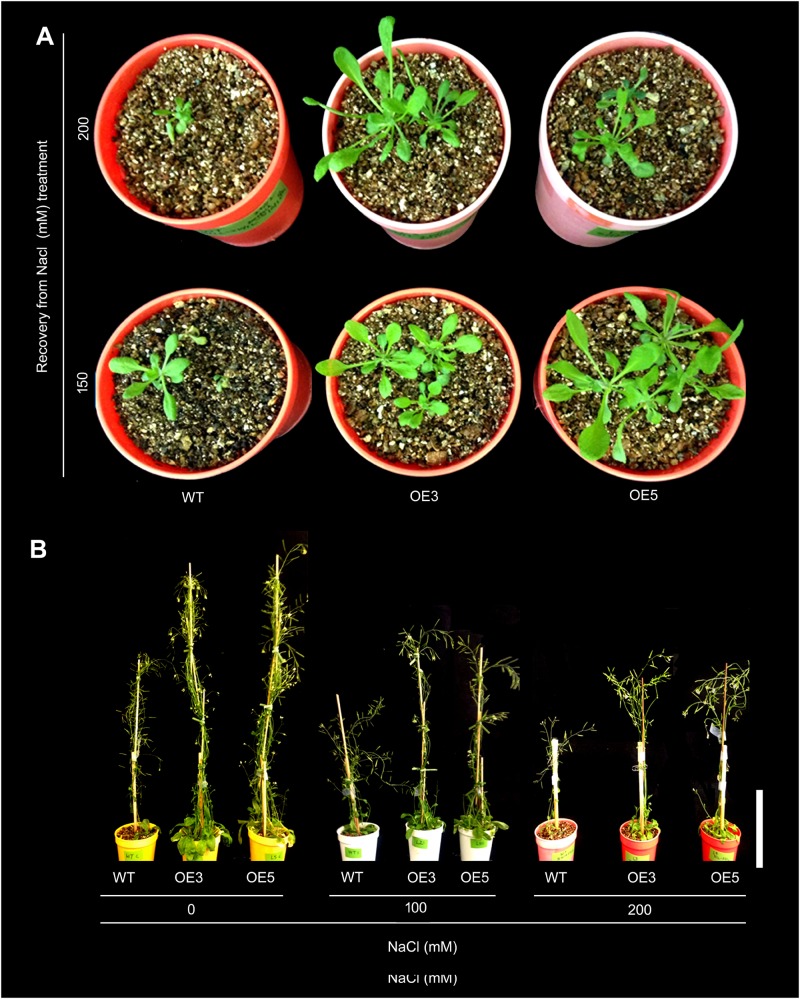
Effect of salt stress on *OsSHMT3* overexpressing Arabidopsis. **(A)** Recovery of wild-type (WT) and *OsSHMT3* overexpressing Arabidopsis (OE3 and OE5) after NaCl stress (150 and 200 mM) and their subsequent transfer to the normal condition for 7 days each. **(B)** WT, OE3 and OE5 were grown for 1 month (up to maturity) in the nutrient solution supplemented with 0 mM (control), 150 and 200 mM NaCl. The experiments were carried out in 3 replicates.

**FIGURE 5:**
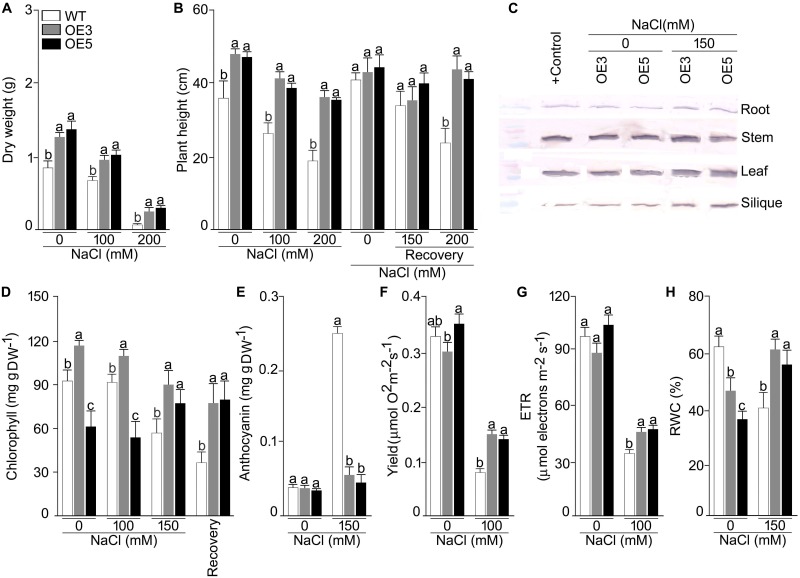
Effects of *OsSHMT3* overexpression in Arabidopsis on morphophysiological, and molecular traits. wild-type and/or Arabidopsis overexpressing *OsSHMT3* (OE3 and OE5) were subjected to NaCl stress and/ or subsequently transferred to the recovery medium for documenting **(A)** dry weight and **(B)** plant height, were calculated relative to the wild type **(C)** OsSHMT3 was used as primary antibody to determine the protein expression of OsSHMT3 in different tissues, **(D)** chlorophyll content, **(E)** anthocyanin content, **(F)** photosynthetic yield, **(G)** ETR, and **(H)** RWC. Values (*n* = 9) are mean ± SE and different letters on the histograms indicate that the values differ significantly (*P* < 0.05). All the data were subjected to two-way ANOVA and significant differences were recorded for within the genotypes and also genotype × environment (salt stress) interactions.

To study the heterologous overexpression of *OsSHMT3* in Arabidopsis in conferring tolerance to the salinity stress, wild-type and *OsSHMT3* overexpressing Arabidopsis (OE3 and OE5) were subjected to salinity stress (150 mM and 200 mM) for 7 d and then transferred to the normal condition for 7 d (Figure 4A). Compared with the wild-type, OE3 and OE5 appeared more robust clearly indicating the efficacy of *OsSHMT3* in conferring tolerance to salinity stress in Arabidopsis. Relatively higher tolerance to salinity stress by OE3 and OE5 compared with the wild-type was also evident when they were grown to maturity (Figure 4B). Overexpressing Arabidopsis, OE3 and OE5 and wild-type were subjected to NaCl stress of 100 and 200 mM for up to 4 weeks and were used to study plant height, dry weight (DW), chlorophyll, anthocyanin, oxygen yield, electron transport rate and relative water content (RWC) (Figure 5A–H). In a recovery experiment wild-type were unable to survive when tried to recover after 200 mM NaCl stress, whereas, recovery after 150 mM NaCl stress showed better performance in growth and seed yield in OE3 and OE5 (Figure 4). Wild-type, OE3 and OE5 were subjected to the salinity stress (100 and 200 mM NaCl) for 4 weeks to determine its effect on the dry weight (Figure 5A) and plant height (Figure 5B). Dry weight and plant height of OE3 and OE5 were significantly higher compared with the wild-type, both under control condition (0 mM NaCl) and during salinity stress (Figure 5A,B). Further, salinity-stressed wild-type, OE3 and OE5 were transferred to the normal medium to determine their recuperation efficacy. Although the recovery of the wild-type and the transgenics (OE3 and OE5) with respect to their plant height was comparable when treated at 150 mM NaCl, only the latter revealed better recuperation ability upon treatment at a higher dosage of salinity stress (200 mM NaCl). The study provided evidence toward better tolerance of OE3 and OE5 to salinity stress compared with the wild-type. The plants were also analyzed for their physiological and biochemical response after salt stress. The total chlorophyll content of wild-type, OE3 and OE5 transgenic Arabidopsis were estimated. The chlorophyll content showed no difference after 100 mM NaCl stress while inhibited significantly after 150 mM NaCl in wild-type and OEs. *OsSHMT3* Arabidopsis could regain its chlorophyll when plants were transferred to normal media for recovery (Figure 5D). Further oxygen yield and electron transport rate was reduced under salt stress in wild-type, OE3 and OE5. However, OE3 and OE5 showed comparatively less reduction in oxygen yield and the electron transport reaction (Figure 5F,G).

Relative water content was significantly reduced in wild-type under NaCl stress condition while OE3 and OE5 showed higher RWC (Figure 5H). To assess the ROS scavenging mechanism, the activities of SOD and POD under salt stress in wild-type and OE3 and OE5 were also analyzed. The SOD activity was significantly (*P* < 0.05) augmented in wild-type but remained the same in OE3 and OE5 as compared with wild-type, whereas, the POD activity was not only higher in wild-type but also in OE3 and OE5 (Supplementary Figures S3A,B) during NaCl stress.

**FIGURE 6:**
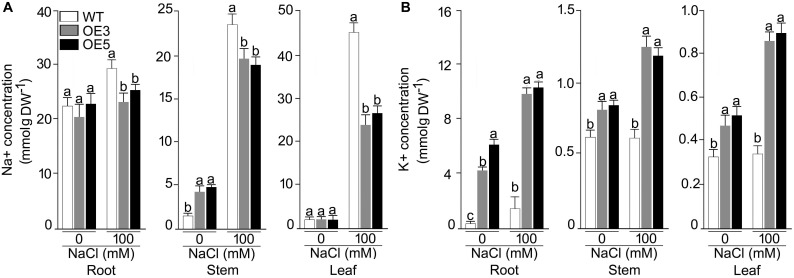
Concentrations of Na^+^, K^+^ in different tissues of the NaCl stressed Arabidopsis overexpressing *OsSHMT3.* The wild-type, OE3 and OE5 seedlings (14-day-old) were subjected to NaCl stress (100 mM) for 1 week, and different tissues (root, stem and leaf) were harvested for determining the concentration of **(A)** Na^+^, and **(B)** K^+^ Values (*n* = 9) are mean ± SE and different letters on the histograms indicate that the values differ significantly (*P* < 0.05). All the data were subjected to two-way ANOVA and significant differences were recorded for within the genotypes and also genotype × environment (salt stress) interactions.

Overexpressers and wild-type were also analyzed for their ionic profiling, and Na^+^ content sharply increased in root, shoot and leaf under NaCl stress in all the plants (Figure 6A,B). Na^+^ accumulation in shoot and leaf were ∼4 and ∼10 fold higher in NaCl (100 mM) stress condition. However, this increase of Na^+^ was less pronounced in OE3 and OE5. A significant (*P* < 0.001) difference in Na^+^ content was observed for all the lines and two-way ANOVA suggested a significant difference at different NaCl levels. K^+^ content was found to be increased in all the lines other than wild-type after NaCl exposure and it reached maximum in root tissues of OE3 and OE5 after NaCl stress.

Amino acid profile of 14-day-old wild-type and transgenic OE3 and OE5 were analyzed after subjecting them for one (data not shown) and 2 weeks of 100 mM NaCl stress. The amino acids for methionine biosynthetic pathway (serine, glycine, cysteine, and methionine) were quantified. In root, shoot and leaf, serine content was significantly higher under NaCl stress condition for wild-type and *OsSHMT3* Arabidopsis. In siliques it was showing a different trend and reduced in OE3 and OE5 after NaCl stress. Glycine was also higher in all the tissues root, shoot, leaf and silique in wild-type, OE3 and OE5 after NaCl stress (Table 1), methionine content showed augmentation in OE3 and OE5 in contrast with wild-type, which did not show any change in its content. A mean comparison suggested a significant difference (*P* < 0.001) for all the amino acids within wild-type, OEs line and also NaCl stress levels.

**Table 1:** Free amino acids in different tissues of the wild-type and *OsSHMT3* overexpressing in *Arabidopsis thaliana* after NaCl (100 mM) stress for 2 weeks.

		Root		Stem		Leaf		Silique	
					
μmol/gFW	Control	NaCl	Control	NaCl	Control	NaCl	Control	NaCl
Serine	wild-type	4.31 ± 0.51	5.88 ± 0.71	4.13 ± 0.51	5.35 ± 0.64	1.06 ± 0.13	8.13 ± 0.97	3.71 ± 0.45	21.27 ± 2.54
	OE3	3.51 ± 0.42	8.13 ± 0.98	4.82 ± 0.61	14.68 ± 1.76	13.35 ± 1.61	6.56 ± 0.78	17.38 ± 2.09	7.25 ± 0.87
	OE5	10.02 ± 1.20	10.21 ± 1.22	8.13 ± 1.01	13.02 ± 1.56	1.68 ± 0.21	0.75 ± 0.09	6.14 ± 0.74	4.14 ± 0.51
Glycine	wild-type	1.13 ± 0.14	3.66 ± 0.44	0.36 ± 0.04	1.89 ± 0.23	0.55 ± 0.07	2.34 ± 0.28	0.64 ± 0.08	3.31 ± 0.41
	OE3	3.22 ± 0.39	3.34 ± 0.40	1.11 ± 0.13	1.28 ± 0.15	1.12 ± 0.13	10.63 ± 1.28	2.79 ± 0.33	12.05 ± 1.41
	OE5	2.75 ± 0.33	1.42 ± 0.17	0.36 ± 0.04	1.01 ± 0.12	2.69 ± 0.32	14.34 ± 1.72	0.77 ± 0.09	10.36 ± 1.24
Cysteine	wild-type	12.91 ± 1.55	15.43 ± 1.85	1.99 ± 0.24	2.31 ± 0.28	0.62 ± 0.07	0.75 ± 0.09	ND	ND
	OE3	0.11 ± 0.01	0.75 ± 0.09	0.36 ± 0.04	1.12 ± 0.13	0.32 ± 0.04	0.74 ± 0.09	ND	ND
	OE5	0.23 ± 0.03	1.44 ± 0.17	1.59 ± 0.19	3.09 ± 0.37	0.37 ± 0.04	0.59 ± 0.07	ND	ND
Methionine	wild-type	0.09 ± 0.01	0.11 ± 0.01	0.22 ± 0.03	0.11 ± 0.01	0.32 ± 0.04	0.02 ± 0.01	0.19 ± 0.02	0.54 ± 0.06
	OE3	0.21 ± 0.03	0.67 ± 0.08	0.11 ± 0.01	0.31 ± 0.04	0.11 ± 0.01	0.05 ± 0.01	0.33 ± 0.04	0.47 ± 0.06
	OE5	0.39 ± 0.05	1.01 ± 0.12	0.06 ± 0.01	0.32 ± 0.04	0.13 ± 0.02	0.05 ± 0.01	0.07 ± 0.06	0.17 ± 0.02


### Gene Expression Profiling in *OsSHMT3* and Wild Type Arabidopsis After Salt Stress

**FIGURE 7:**
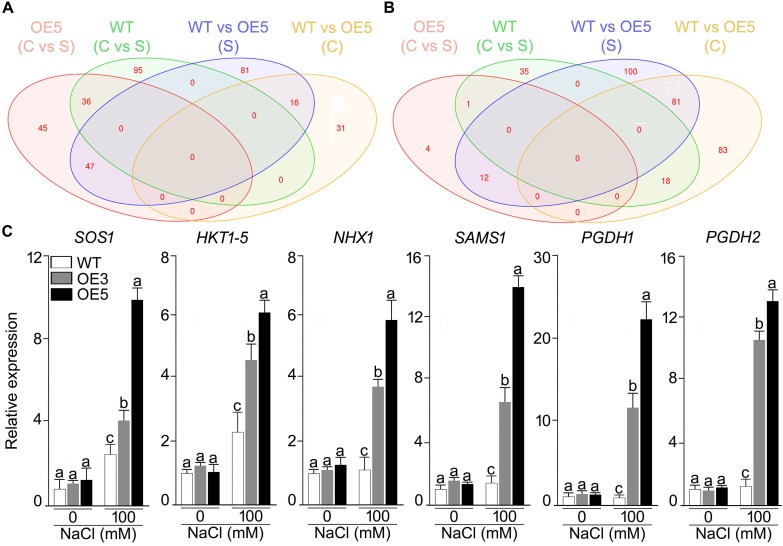
Effect of salt stress on the gene expression profiling of wild-type and Arabidopsis overexpressing *OsSHMT3*. Seedlings (14-day-old), subjected to 100 mM NaCl stress for 24 h, were used for RNA extraction. Venn diagram showing the genes in wild-type and OE5 that are **(A)** up-regulated, and **(B)** down-regulated. **(C)** Seedlings (14-day-old) of the wild-type, OE3 and OE5 were subjected to NaCl (100 mM) stress for 24 h, and the whole seedlings were used for assaying the relative expression of the salt stress responsive genes. *Actin* was used as an internal control. Values (*n* = 9) are mean ± SE and different letters on the histograms indicate that the values differ significantly (two-way ANOVA; *P* < 0.05).

Whole genome expression profiling was done for wild-type and *OsSHMT3* Arabidopsis after NaCl stress (150 mM). In total 9401 gene probes in wild-type and 2840 in OE5 were significantly expressed, among which 1956 were common to wild-type and OE5 (Figure 7). After applying NaCl stress, 167 genes in wild-type and 130 genes in OE5 were differentially expressed, and among them 52 were common to both when compared with their controls. Differentially expressed genes belonged to different functional categories (Supplementary Table S1). Among transcription factors, homeobox12 (ATHB-12) RAP2.6 (related to AP2 6), NAC domain containing protein and ethylene-responsive transcription factor (ERF054) were upregulated in OE5. However, a greater number of TFs were upregulated in wild-type containing MYBs and WRKYs. Arabidopsis homeobox 12 (ATHB12) is a transcription factor, which was up-regulated in *OsSHMT*3 Arabidopsis. Ethylene-responsive transcription factor (ERF) was found to be up-regulated by 2.4-fold in *OsSHMT3* Arabidopsis during the seedling stage. Proline dehydrogenase 2, peroxidases, glyoxlase, glutathione s-transferase and Late Embryogenesis Abundant (LEA) family protein were all upregulated in OE5 after salt stress. LEA family proteins were induced in both *OsSHMT3* over-expressers and wild-type Arabidopsis. The water channel proteins like plasma membrane intrinsic protein (PIP2;5); beta-tonoplast intrinsic protein (BETA-TIP and TIP3;1), sulfate transporter (SUL3;1), nitrate transporters and bidirectional sugar transporter (SWEET4) were expressed only in OE5 after NaCl stress. In this study, expression of SWEET transporter was reported for salt stress for the first time. Nitrate transporter 1 (NPF2.4), peptide transporter family genes were induced in *OsSHMT3* Arabidopsis after salt stress. Since SHMT belongs to a biochemical synthetic pathway of methionine biosynthesis genes, some of these genes were also found to be expressed in OE5 like; homocysteine S-methyltransferase (HMT3) and S-adenosylmethionine-dependent methyltransferase, which were upregulated in OE5 after NaCl stress.

Among the over-expresser (OE3 and OE5) transgenic lines, some of signature genes of salt stress were found to express differentially under NaCl stress, as analyzed by RT-qPCR (Figure 7C). HKT 1;5, SAMS, PGDH1, and PGDH2 showed higher expression in 100 mM NaCl stress as compared with its control in both OE3 and OE5. PGDH1 and PGDH2 showed 20- and 12-fold higher mRNA expression in OE3 and OE5, respectively. Over all *OsSHMT3* transgenic lines exhibited higher expression of all the six signature genes identified for salt stress tolerance. HKT1;5 and SAMS depicted 5- and 12-fold higher expression in OE5. The higher expression of these genes indicated salt-tolerant nature of *OsSHMT3* Arabidopsis.

## Discussion

The study aimed to decipher the role of *OsSHMT3* in conferring tolerance in heterologous *E. coli* and Arabidopsis. In *A. thaliana*, seven SHMT genes have been identified (*AtSHM1-7*) (McClung et al., 2000), of which localization of *AtSHM1* (At4g37930) and *AtSHM*2 (At5g26780) in mitochondria (Jamai et al., 2009; Engel et al., 2011), *AtSHM3* (At4g32520) in plastids (Zhang et al., 2010) and *AtSHM4* (At4g13930) in cytosol (Wei et al., 2013), have been demonstrated. However, subcellular localizations of other members [*AtSHM5* (At4g13890), *AtSHM6* (At1g22020), and *AtSHM7* (At1g36370)] have not yet been reported. *AtSHM2* could not complement the mutation in *AtSHM1*, suggesting then to be functionally non-redundant (Engel et al., 2011). Consistent with these studies, the present study also revealed a tissue-specific differential expression pattern of *OsSHMT1-5* in both salt-tolerant (CSR27) and salt-sensitive (MI48) rice genotypes (Figure 1A). Since the relative expression of *OsSHMT3* was significantly higher in CSR27 in both shoot and root, it suggested a possible role of this member of the family in conferring tolerance toward salinity stress.

An earlier study also identified several molecular entities belonging to different functional categories that were differentially expressed in these two contrasting genotypes (Pandit et al., 2010). This suggested that *OsSHMT3* and perhaps some other members of this family may have a role in intricate sensing and signaling cascade that confers tolerance toward salinity. Generally, plant responses to salinity stress have been classified into “osmotic,” “ionic-specific,” and the subsequent effects (oxidative effects) (Munns and Tester, 2008; Roy et al., 2014; Arzani and Ashraf, 2016). Normally, during the early stage of salinity, when accumulation of salts in plant roots is low, hyperosmotic is the main deleterious effect. The long-term effects include excessive salt uptake, which later cause the plants to suffer from the toxic effects of extra Na^+^ and Cl^+^ ions. The Na^+^ and Cl^+^ ions are taken up by the roots and then translocated to aerial organs, where they can be accumulated. Excessive Na^+^ accumulation adversely affects the cell metabolism and physiological operations and could even lead to cell death when ion levels rise to a certain level (Hasegawa, 2013). In-depth illustrations of the salinity-induced osmotic and ionic stresses have been described in this review and elsewhere (Munns and Tester, 2008; Hasegawa, 2013; Arzani and Ashraf, 2016). Salinity stress for 24 h triggered significant changes in both the phenotype of the seedlings and differential regulations of an array of genes in them. Salt stress regulates spatiotemporal transcriptional responses in plant, which may have role in water transport and protein translational machinery (Geng et al., 2013). The expression of the DREB gene from *Solanum lycopersicum* (*SlDREB2*) showed an increase in the expression until 12 h after onset of salinity and then reduced at 24 h in young leaves of Arabidopsis (Hichri et al., 2016). This correlates well with the expression of *OsSHMT3* which depicted a similar trend of expression during NaCl stress.

Further, the *SHMT3* gene was cloned from salt-tolerant rice and expressed in a protein expression vector for detailed analysis. The amino acid sequence homology of OsSHMT3 showed its similarity to *A. thaliana* AtSHMT1 and AtSHMT2, and human (Renwick et al., 1998; Wei et al., 2013). Based on the similarity, the PDB based X-ray resolved structure was drawn which confirmed the stability of SHMT proteins because of formation of salt bridges and intact functional residues. However, the OsSHMT3 protein was expressed in inclusion bodies, which made the protein insoluble and inactive (Mayer and Buchner, 2004). Therefore, the functional conformation for enzyme was recovered by affinity tagging after addition of consecutive histidine residues. The enzyme assays with substrates serine and THF followed standard enzyme kinetic and indicated active functional state of enzyme. SHMT is abundant in nature and has a pivotal role in one-carbon folate metabolism and acts as a core regulator for Ser↔Gly conversion in seeds of *Solanum pennillii* (Toubiana et al., 2015). It has multiple catalytic activities to catalyze the reversible conversion of serine and THF to glycine and MTHF, providing one-carbon units for methionine synthesis and methylation reactions (Liu et al., 2012). The SHMT of soybean (Mitchell et al., 1986) is a tetramer, with an optimum pH 8.5, that showed 2 Km values for serine at 1.5 and at 40 mM. Salinity-stressed *E. coli* overexpressing *OsSHM3* revealed significantly higher levels of metabolities (glycine, serine, and methionine). Also in earlier studies, a higher activity of ApSHMT in marine cyanobacterium was found to concur with elevated levels of glycine (Waditee-Sirisattha et al., 2012, 2017). Since serine and glycine are the upstream metabolites for glycine betaine, the GB and choline content were also enhanced after NaCl stress in *OsSHMT3* overexpressing *E. coli*, as reported for *ApSHMT* (Waditee-Sirisattha et al., 2012). Glycine betaine is one of the important osmolytes, which protects the plants from salt stress. Overexpression of glycine betaine synthesis genes from marine cyanobacterium (*Aphanothece halophytica*) to *A. thaliana*, leads to a higher level of GB and better seed yield (Waditee et al., 2005) under salt stress condition.

Further *OsSHMT3* was overexpressed in *A. thaliana*, which demonstrated its tolerant behavior toward salt stress. The plant height of two independent overexpressing lines (OE3 and OE5) were always higher than wild-type under salt stress, as reported for *Brassica* where salinity leads to reduction of plant height, size and yield (Zamani et al., 2010). Salinity-induced decrease in chlorophyll was lower in the *OsSHMT3* plants, in comparison with wild-type. Salinity stress induced bleaching of leaf tissues in the wild-type indicating its adverse effect on photosynthesis. This observation was consistent with an earlier study reporting salinity stress-induced significant reduction in both the oxygen yield and electron transport reaction in Arabidopsis (Stepien and Johnson, 2009). On the contrary, no apparent bleaching of leaf tissues could be detected in either OE3 or OE5. This suggested the ability of OE3 and OE5 to continue normal photosynthetic activity despite being subjected to salinity stress. Higher RWC in the transgenics (OE3 and OE5) compared with the wild-type after recuperation from the salinity stress further provided evidence toward the efficacy of the former in maintaining its internal turgor pressure. When Arabidopsis was transformed with Myb transcription factor family gene from *Gossypium arboreum* (*GaMYB85*), the RWC in transgenic lines showed lesser reduction as compared with wild-type plant (Butt et al., 2017) during osmotic stress. SbSRP gene from *Salicornia brachiata* grown in salt marshes and Osmotin-like proteins (OLPs) from sesame (*SindOLP*) increased the RWC after salt stress (Udawat et al., 2017). Higher SOD and POD activity in transgenic lines showed its tolerant nature against salt stress as reported (Sun et al., 2017). SOD and POD are antioxidant enzymes, which help in reactive oxygen species scavenging mechanisms by increasing its concentration, and thus provide stress tolerance in plants. Salinity stress adversely affects osmotic balance of the plants and induces ion toxicity. Balance of Na^+^ and K^+^ is a prerequisite for detoxification of plant from excess Na^+^ (Deinlein et al., 2014). In tomato plant, *CodA* gene overexpression leads to lower Na^+^ and higher K^+^ accumulation, in comparison with wild-type (Wei et al., 2017) which corresponds to the present study where *OsSHMT3*-overexpressed Arabidopsis showed lesser Na^+^ and higher K^+^ content after salt stress in different tissues. Na^+^ exclusion if combined with tissue tolerance would be more beneficial for salt tolerance (Shabala et al., 2013). K^+^ retention in the cell is one of the components for tissue tolerance and supplementing K^+^ fertilizers can enhance the salt tolerance of plants (Shabala and Pottosin, 2014). A significant positive correlation was reported for retaining of K^+^ under salt stress condition (Wu et al., 2013) for salt-tolerance. Based on the ability of plant to discriminate the potential between K^+^ and Na^+^ in the soil solution, as well as preferably exclude Na^+^ and accumulate K^+^ from metabolically active tissues, the K^+^:Na^+^ discrimination has been considered as a critical salt tolerance mechanism in plant species (Arzani and Ashraf, 2016). The existence of multiple channel types with different selectivity’s for K^+^:Na^+^ might reflect a coordinate regulation of the influx of these two ions. Nonetheless, the regulation process of these non-selective cation channels (NSCCs) and signaling pathways, that activate the genes involved in non-selective channels, are yet unknown. Moreover, lowering of K^+^ content under salt stress is primarily due to lesser uptake of K^+^ from external medium because of increased competition with Na^+^ in a salty environment and loss of K^+^-retention capacity in different plants as a result of increased K^+^ leakage by voltage or ROS gated K^+^-channels (Wu et al., 2014). Demidchik et al. (2014) suggested that the main consequence of electrolyte leakage is stress-induced K^+^ release, which caused by outwardly rectifying K^+^ channels activated by ROS in plant cells. The K^+^ loss resulting from ion channel channel-mediated K^+^ efflux can induce PCD (Demidchik et al., 2014). The phenomenon of ROS generation, leading also to PCD, is not an independent process but may largely be influenced by the K^+^ loss in conditions of stress-induced electrolyte leakage. In plant cells highly selective outward rectifying K^+^ channel (SKOR), guard cells outward rectifying K^+^ channel (GORK) and annexins catalyzing K^+^ influx can be activated by ROS (OH and H_2_O_2_). In addition, under salinity and oxidative stress, PCD can be induced by GORK-mediated K^+^ efflux (Demidchik et al., 2014). Higher leaf K^+^ content was reported for bread wheat which was more salt tolerant than durum wheat (Wu et al., 2014), suggesting that a higher level of K^+^ is one of the components for higher salt tolerance in *OsSHMT3* Arabidopsis. Salinity stress is known to have significant effects on the levels of various amino acids (Sanchez et al., 2008). In fact, salinity stress-mediated augmented levels of serine have correlated with an elevated photorespiration (Hossain et al., 2017). In the present study, the serine content was relatively lower in the leaf tissues of Arabidopsis transgenics (OE3 and OE5), compared with the wild-type. Therefore, photorespiration in the transgenics could be assumed to be relatively lower than the wild-type. The higher level of glycine and serine may have a role in maintaining osmotic potential and thereby protecting damage of the cell by high Na^+^/Cl^-^ ions. To study the changes in gene expression, whole genome expression profiling was employed which leads to identification of differentially expressed genes in *OsSHMT3* Arabidopsis. ATHB12 is a homeodomain-leucine zipper class I (HD-Zip I) gene, which was highly expressed in leaves and stems, and induced by abiotic stresses (Hur et al., 2015). ERF is a conserved, super family transcription factor which was highly induced in salt stress in OE5. It plays crucial roles in plant development, response to biotic and abiotic stresses and programmed cell death (Bahieldin et al., 2016). AP2/ERF proteins played important roles in diverse biological activities. According to Zhu et al. (2010), RAP2.6 is involved in abiotic stress, likely through the ABA-dependent pathway. NAC transcription factors form one of the biggest groups of transcriptional controllers in the plant system, which play a vital role in gene regulation, especially transcriptional reprogramming in stress conditions (Nuruzzaman et al., 2013). SlNAC8, a transcription factor from the halophyte *Suaeda liaotungensis*, regulates the stress responsive genes and augmented the salt stress tolerance of Arabidopsis (Wu et al., 2018). The overexpression of ethylene response transcription factor (AhERF) from *Amaranthus hypochondriacus* improved Arabidopsis tolerance to water-deficit stress without any negative impact on plant growth (Massange-Sánchez et al., 2016). The salt tolerance of OsSHMT3 Arabidopsis may be due to cumulative effects of the higher expression of NAC and ERF transcription factors. Further, microarray and real time PCR analyses of the wild-type and transgenic Arabidopsis overexpressing *OsSHMT3* revealed a significantly higher expression of an array of genes, including those encoding for aquaporin, chloride transporter, HKT1-5, NHX1, osmotin, PGDH1 and 2, SAM SOS1 and SWEET proteins in the latter compared with the former (Figure 7 and Supplementary Table S1). The analyses highlighted the potential key roles of these genes in exerting their varied influence on the cascade of reactions that govern responses to salinity stress. The results were coherent with several earlier studies implicating the roles of these genes in salinity stress responses in diverse plant species [aquaporin (Siefritz et al., 2002; Hachez et al., 2012, 2014; Pou et al., 2016), chloride transporter (Li et al., 2016), HKT1-5 (Kobayashi et al., 2017), NHX1 (Barragan et al., 2012), osmotin (Kumar et al., 2014), PGDH1 and 2 (Benstein et al., 2013; Kito et al., 2017), SAM (Kim et al., 2015), SOS1 (Yang et al., 2009), and SWEET proteins (Chong et al., 2014)]. Overall the study revealed the role of *OsSHMT3* during salinity stress through exerting an influence on aquaporins in maintaining the turgor balance and mitigating the ion toxicity effects via HKT1; 5, NHX1 and chloride transporters.

In conclusion, the detailed morphophysiological and molecular analyses empirically revealed the efficacy of *OsSHMT3* in conferring tolerance to salinity stress, upon heterologous overexpression in diverse *E. coli* and Arabidopsis. This suggested a possible overlapping and pivotal role of this gene in intricate sensing and signaling cascades that govern salinity stress responses in these organisms. Further, microarray analysis highlighted significant increases in the expression levels of several genes belonging to different functional categories, including aquaporins in the Arabidopsis overexpressing *OsSHMT3* compared with the wild-type. Further studies are warranted for more in-depth studies on the effects of *OsSHMT3* on genes encoding aquaporins and/or other molecular entities that may have a role in conferring tolerance to salinity stress in taxonomically diverse species. Emerging state-of-the-art genome editing technologies provide an attractive paradigm to achieve the said objectives.

## Author Contributions

NKS and VR conceived and designed the work. PM, NS, NJ, VM, RM, and MN performed the experiments. TT, YT, SK, and RS helped and analysis of results. VR and AJ wrote the manuscript.

## Conflict of Interest Statement

The authors declare that the research was conducted in the absence of any commercial or financial relationships that could be construed as a potential conflict of interest.

## Footnotes

1 www.fao.org/publications/sofi/

2 https://esa.un.org/unpd/wpp

3 www.faostat.fao.org/

4 www.Arabidopsis.org/

5 www.tigr.org/

6 www.ncbi.nlm.nih.gov/

7 www.sanger.ac.uk/Software/Pfam/search.shtml
