# Supplementary material for: Heterologous Expression of Serine Hydroxymethyltransferase-3 From Rice Confers Tolerance to Salinity Stress in E. coli and Arabidopsis
Source: Front Plant Sci. 2019 Mar 19;10:217. doi: 10.3389/fpls.2019.00217 (PMC6433796; doi:10.3389/fpls.2019.00217)
Supplement: Supplementary file 1 [file Table_1.DOC]

**SUPPLEMENTARY INFORMATION**

**Supplementary Table 1**: Highly induced gene in *OsSHMT* overexpressing and wild type Arabidopsis after NaCl stress. All genes were highly significant with log fold change >2 at p<0.001.

| **Category** | **Gene id** | ***OsSHMT* Arabidopsis** | **Wild Type** |
| --- | --- | --- | --- |
| **Transcription factors**  **(6)** | ATHB-12 | ATHB-12 (ARABIDOPSIS THALIANA HOMEOBOX 12); transcription activator/ transcription factorHighly | ATHB-12 (ARABIDOPSIS THALIANA HOMEOBOX 12); transcription activator/ transcription factor |
|  | AT4G28140 | ethylene-responsive transcription factor ERF054 |  |
|  | anac032 | anac032 (Arabidopsis NAC domain containing protein 32); transcription factor | ANAC13 (Arabidopsis thaliana NAC domain protein 13); transcription factor |
|  | RAP2.6 | RAP2.6 (related to AP2 6); DNA binding / transcription factor | RAP2.6 (related to AP2 6); DNA binding / transcription factor |
|  |  |  | AtMYB74; AtMYB102 (myb domain protein 74); DNA binding / transcription factor |
|  | WRKY |  | AT-HSFC1; AT-HSFA6A; DNA binding / transcription factor |
|  |  |  | WRKY6; WRKY28; transcription factor |
| **Defense related**  **(17)** | AT5G38710 | proline dehydrogenase 2 |  |
|  | LEA4-5 | late embryogenesis abundant protein 4-5 | late embryogenesis abundant protein 4-5 |
|  | AT1G32560 | Late embryogenesis abundant protein, group 1 protein | Late embryogenesis abundant protein (LEA) family protein |
|  | AT3G17520 | late embryogenesis abundant protein (LEA) family protein | Late embryogenesis abundant protein (LEA) family protein |
|  | AT3G02480 | Late embryogenesis abundant protein (LEA) family protein | late embryogenesis abundant domain-containing protein |
|  | AT1G52690 | Late embryogenesis abundant protein (LEA) family protein | late embryogenesis abundant protein 4-2 |
|  | AT2G42560 | late embryogenesis abundant domain-containing protein |  |
|  | LEA | LEA (DEHYDRIN LEA) |  |
|  | AT2G38380; AT2G38390 | peroxidase 22; peroxidase 23 |  |
|  | AT5G05340 | peroxidase 52 |  |
|  | AT1G80160 | GLYOXYLASE I 7 |  |
|  | LTI30 | LTI30 (LOW TEMPERATURE-INDUCED 30) |  |
|  | AT2G38905 | Low temperature and salt responsive protein |  |
|  | ATGSTU4 | ARABIDOPSIS THALIANA GLUTATHIONE S-TRANSFERASE TAU 4; glutathione transferase |  |
|  | ATGSTU6 | ARABIDOPSIS THALIANA GLUTATHIONE S-TRANSFERASE TAU 6; glutathione transferase |  |
|  | ATGSTU24 |  | GLUTATHIONE S-TRANSFERASE TAU 24; glutathione binding / glutathione transferase |
|  | ATOSM34 |  | Osmotin 34 |
| **Transporters**  **(7)** | PIP2;5 | PLASMA MEMBRANE INTRINSIC PROTEIN 2;5; water channel |  |
|  | BETA-TIP; AT1G17810, PIP2;8 | BETA-TONOPLAST INTRINSIC PROTEIN; water channel |  |
|  | TIP3;1 | water channel |  |
|  | SULTR3;1 | SULFATE TRANSPORTER 3;1; secondary active sulfate transmembrane transporter/ sulfate transmembrane transporter/ transporter |  |
|  |  | nitrate transporter 1.3 |  |
|  | ATNPF7.2, | nitrate transporter 1.8; NITRATE TRANSPORTER 1.8, |  |
|  | AT3G21670 | bidirectional sugar transporter SWEET4 | bidirectional sugar transporter SWEET2 |
|  |  |  | transmembrane amino acid transporter family protein |
|  | ATSUC7 |  | Sucrose-proton symporter 7; carbohydrate transmembrane transporter/ sucrose:hydrogen symporter/ sugar:hydrogen symporter |
| **Meth Biosynthesis**  **(5)** | CORI3 | CORONATINE INDUCED 1; cystathionine beta-lyase/ transaminase |  |
|  | GAPC1 | GLYCERALDEHYDE-3-PHOSPHATE DEHYDROGENASE C SUBUNIT 1; glyceraldehyde-3-phosphate dehydrogenase (phosphorylating) |  |
|  | HMT3 | HMT3; homocysteine S-methyltransferase |  |
|  | BSMT1 | S-adenosylmethionine-dependent methyltransferase | S-adenosylmethionine-dependent methyltransferase |
|  | XPL1 |  | XIPOTL 1 methyltransferase/ phosphoethanolamine N-methyltransferase |

**Supplementary Table 2**: Primers used in the study

| Purpose | Sequence |  |
| --- | --- | --- |
|  |  |  |
| **Primer for RT(Rice)** | **Forward:** TATGTCACCTGGATTCAA  **Reverse:** CAAGATGGTTCTCTGTTC | **SHMT1**  LOC_Os12g22030 |
|  | **Forward:** CAGCACATATAGCAAGTAT  **Reverse:** TATTGTTCTCCAGACCTT | **SHMT2**  LOC_Os05g35440 |
|  | **Forward:** TACATCAACTACTCACAAG  **Reverse:** GAAGTCATAATCATTCTCATC | **SHMT3**  LOC_Os01g65410 |
|  | **Forward:** TGGCAAGTATCTTATGAG  **Reverse:** GCATTCTTGTTAAGTGTAA | **SHMT4**  LOC_Os11g26860 |
|  | **Forward:** TCCTGGTGATGTTTCAGCTATG  **Reverse:** CCTTAGCAAAGTCCTCCTCAAC | **SHMT5**  LOC_Os03g52840 |
| **Primer for cloning of full lengthSHMT3 in pET vector** | **Forward:** EcoRI : 5'- AAA AAG AAT TCA TGG CCA TGG CGA CGG CG -3'  **Reverse:** XhoI : 5'- AAG GCT CGA GGT TCT TGT ACT TCA TGG TTT CTT TCT C -3' | **SHMT3**  LOC_Os03g52840 |
| **Primer for cloning of full lengthSHMT3 in pORE vector** | **Forword:** XbaI: 5'- GCTCTAGAATG GCC ATG GCG ACG GCG CTC C -3  **Reverse:**SalI: 5'- ACGCGTCGACTTA GTT CTT GTA CTT CAT GGT TTC TTT CTC -3' | **SHMT3**  LOC_Os03g52840 |
| **Southern Hybrididation** | **Forward:** TGAAAGCACTGGCTTGATTG  **Reverse:** CTGCATCGAAGAAATCAGCA | **SHMT3**  LOC_Os03g52840 |
| **nptII** | **Forward:** AAGGCCGGCCATGATTGAACAAGATGGATTGCACGC  **Reverse:** AAGGCGCGCCTCAGAAGAACTCGTCAAGAAGGCG |  |
| **Primer for RT(Arabidopsis)** |  |  |
|  | **Forward:** CCAACTGGAGTCTGGCTTATT  **Reverse:** CCCAATGTACTACCGTGAGAAA | **SOS1** |
|  | **Forward:** CGGTGCTAGGGTTTCTGATAAT  **Reverse:** CGATCCAACCATCTCGT | **HKT1;5** |
|  | **Forward:**  GGAAGGGACGATGCTGATTT  **Reverse:**  CTGGACAGAAGTGAAACAGAGG | **SAMS3** |
|  | **Forward:** CACTCGAACCGTGCATTACTA  **Reverse:** TGGAGAACCTGGAACAAAGG | **NHX1** |
|  | **Forward:** AGAGCCCATTGGAGACAATAAC  **Reverse:** GGGACTCCGTCTTTAACCTTTC | **PGDH1** |
|  | **Forward:** CTGAGGTCTTGTCTGAGTTGAC  **Reverse:**  TGACCGATAGACCACCCTAAT | **PGDH2** |

**SUPPLEMENTARY FIGURES**

**Supplementary Figure S1.** Effect of salinity stress on the relative expression levels of *OsSHMT3* in contrasting sensitive and tolerant rice cultivars. Seedlings (14-d-old) were subjected to salinity stress (150 mM NaCl) for 24 h. Real-time PCR analyses were carried out for determining the relative expression levels in shoot and root. Data (*n*=6) represented values that were generated from two independent biological replicates with 3 technical replicate each. Significant difference between sensitive and tolerant rice cultivars is indicated with an asterisk (*P*<0.05), oneway ANOVA.

**Supplementary Figure S2.** Phylogenetic relationship of OSSHMTs with lower and/or higher plant species. **(A)** The amino acid sequences of OsSHMT1-5 were aligned with AtSHMT1 by using CLUSTAL Omega (V. 1.2.0). **(B)** The phylogenetic tree was constructed with neighbor-joining method using full length sequences from *Aphanothece halophytica (ApSHMT), E. coli (EcSHMT), Neurospora crassa (NcSHMT), Saccromyces cerevisiae (ScSHMT), Arabidopsis thaliana (AtSHMT), Oryza sativa (OsSHMT), Zea mays (ZmSHMT), Glycine max (GmSHMT), Pisum sativum (PsSHMT). Triticum aestivum. (TaSHMT), Homo sapiens (HsSHMT), Solanum tuberosum (StSHMT).* The bar represents the genetic distance in the phylogenetic tree.

**Supplementary Figure S3.** Effect of salinity on reactive oxygen scavenging enzyme activities **(A)** SOD and **(B)** POD of WT and *OsSHMT3* overexpressed (OE) Arabidopsis. Seedlings (14-d-old) were transferred to 150 mM NaCl for one week and enzymes were extracted from the leaves. Data were presented for *n*=9 from three biological replicates. Values (*n* = 9) are means ± SE and different letters on the histograms indicate means that differ significantly (*P*<0.05). All the data were subjected to two-ways ANOVA.

**Supplementary Figure S4.** Work flow for the overexpression of *OsSHMT3* in *E. coli* and *A. thaliana*. **(A)** gene construct **(B)** salt-sensitive and salt-tolerant rice cultivars subjected to salt stress (150 mM NaCl) for 24 h. **(C)** PCR of amplification of *OsSHMT3* **(D)** confirmation of *OsSHMT3* by restriction digestion **(E)** selection of the Arabidopsis transgenics **(F)** growth of T1 generation plants.
