# Supplementary figures and images for: Heterologous Expression of Serine Hydroxymethyltransferase-3 From Rice Confers Tolerance to Salinity Stress in E. coli and Arabidopsis
Source: Front Plant Sci. 2019 Mar 19;10:217. doi: 10.3389/fpls.2019.00217 (PMC6433796; doi:10.3389/fpls.2019.00217)

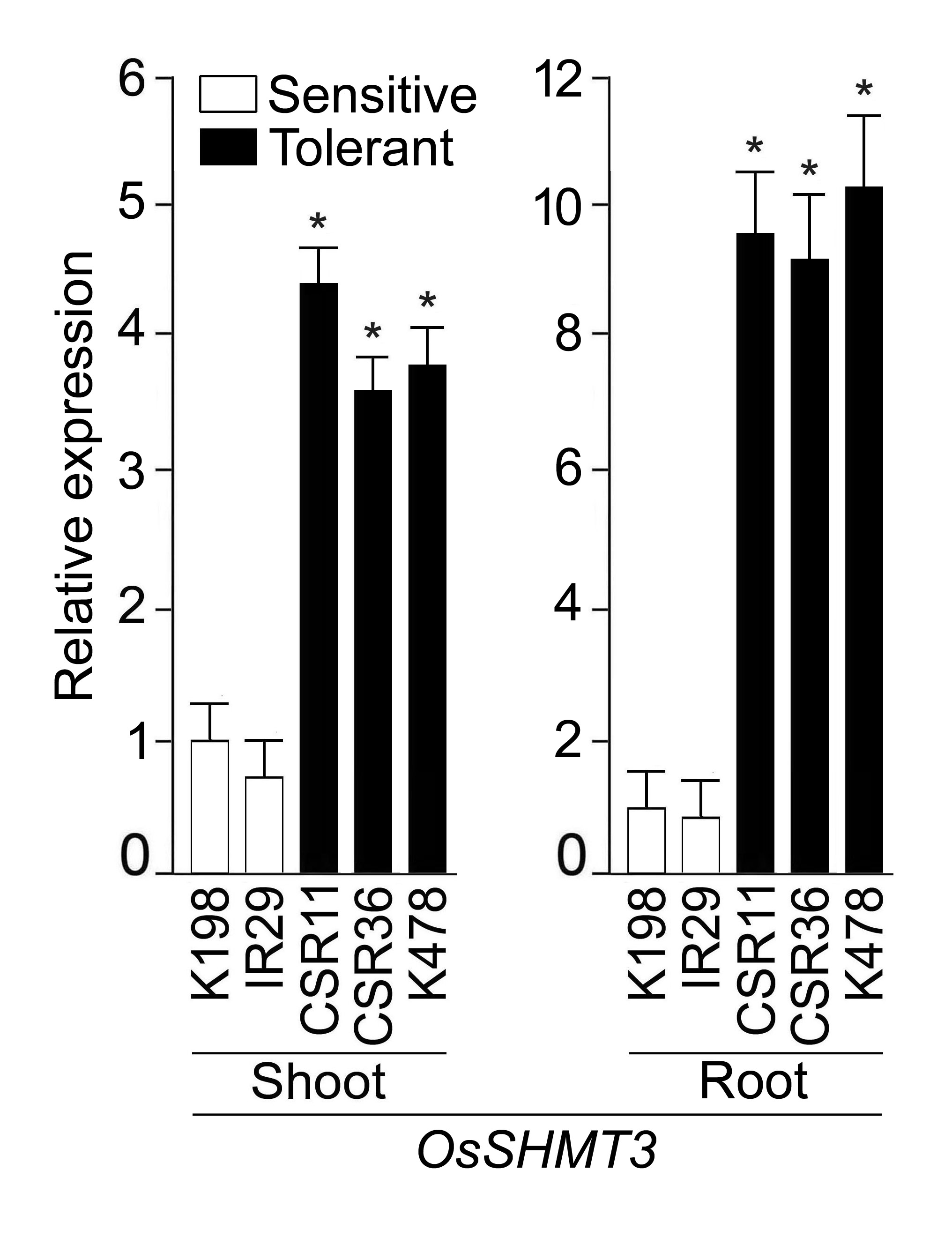

Supplement: Supplementary file 2 [file Image_1.JPEG]

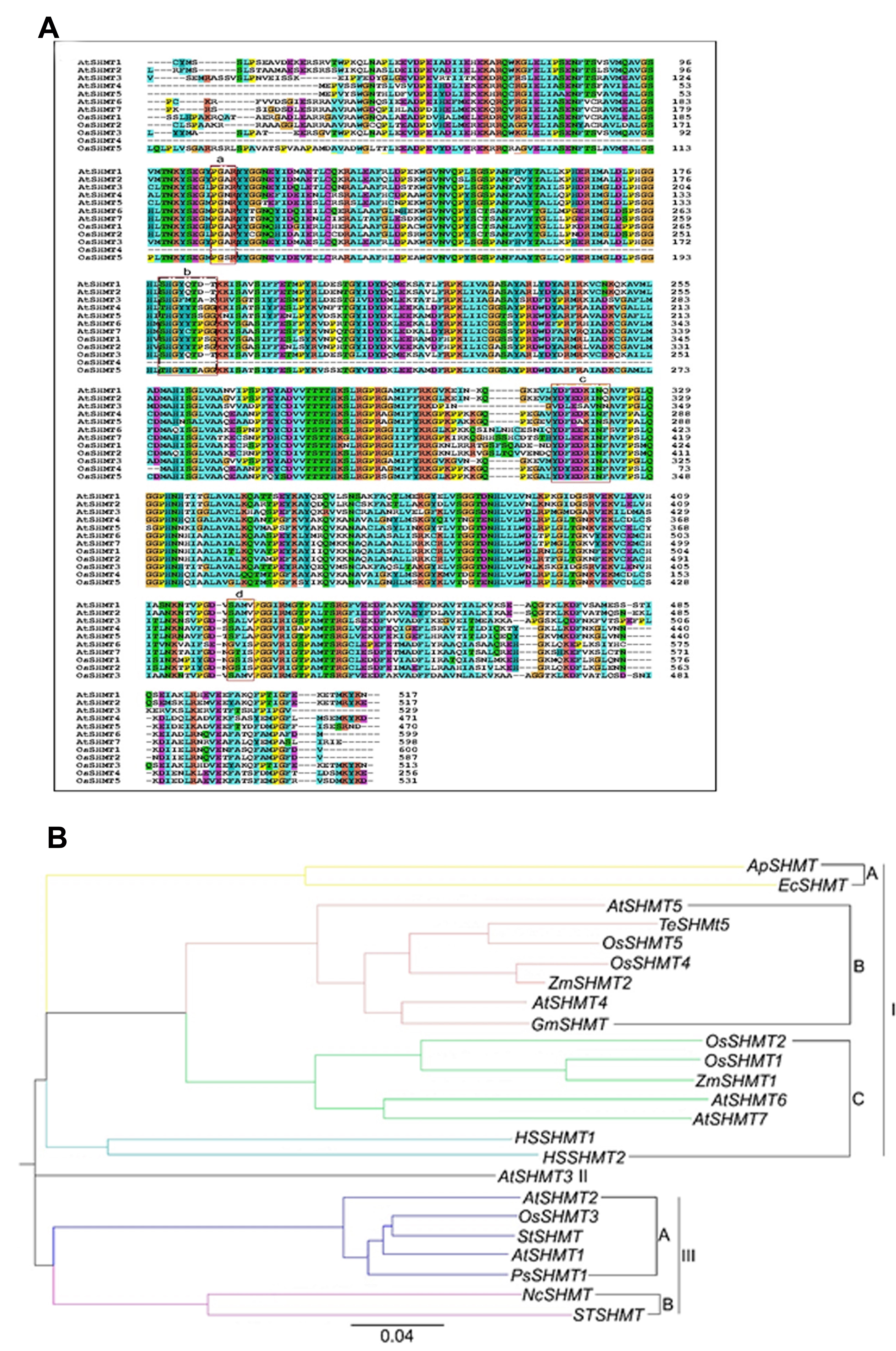

Supplement: Supplementary file 3 [file Image_2.jpg]

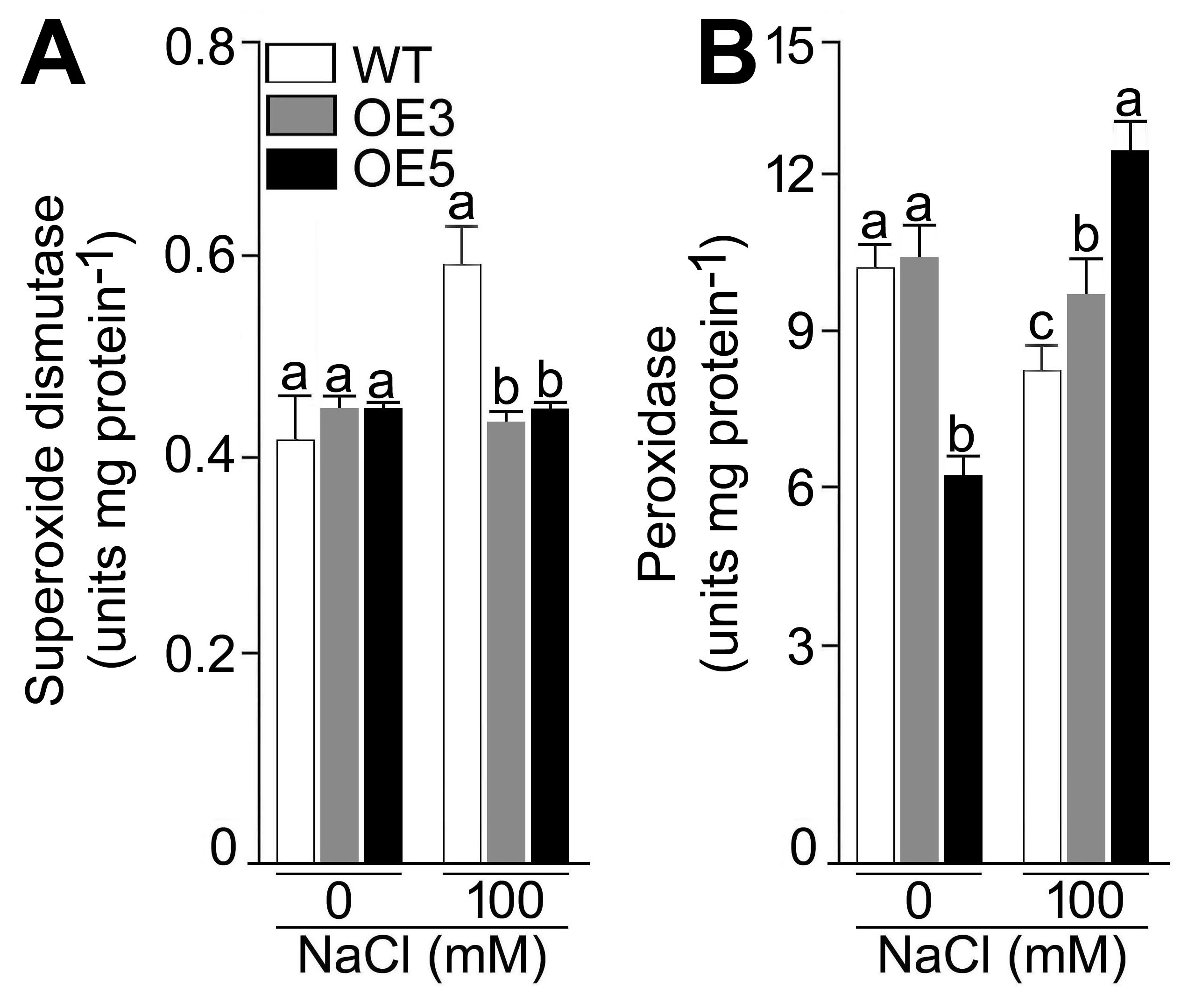

Supplement: Supplementary file 4 [file Image_3.JPEG]

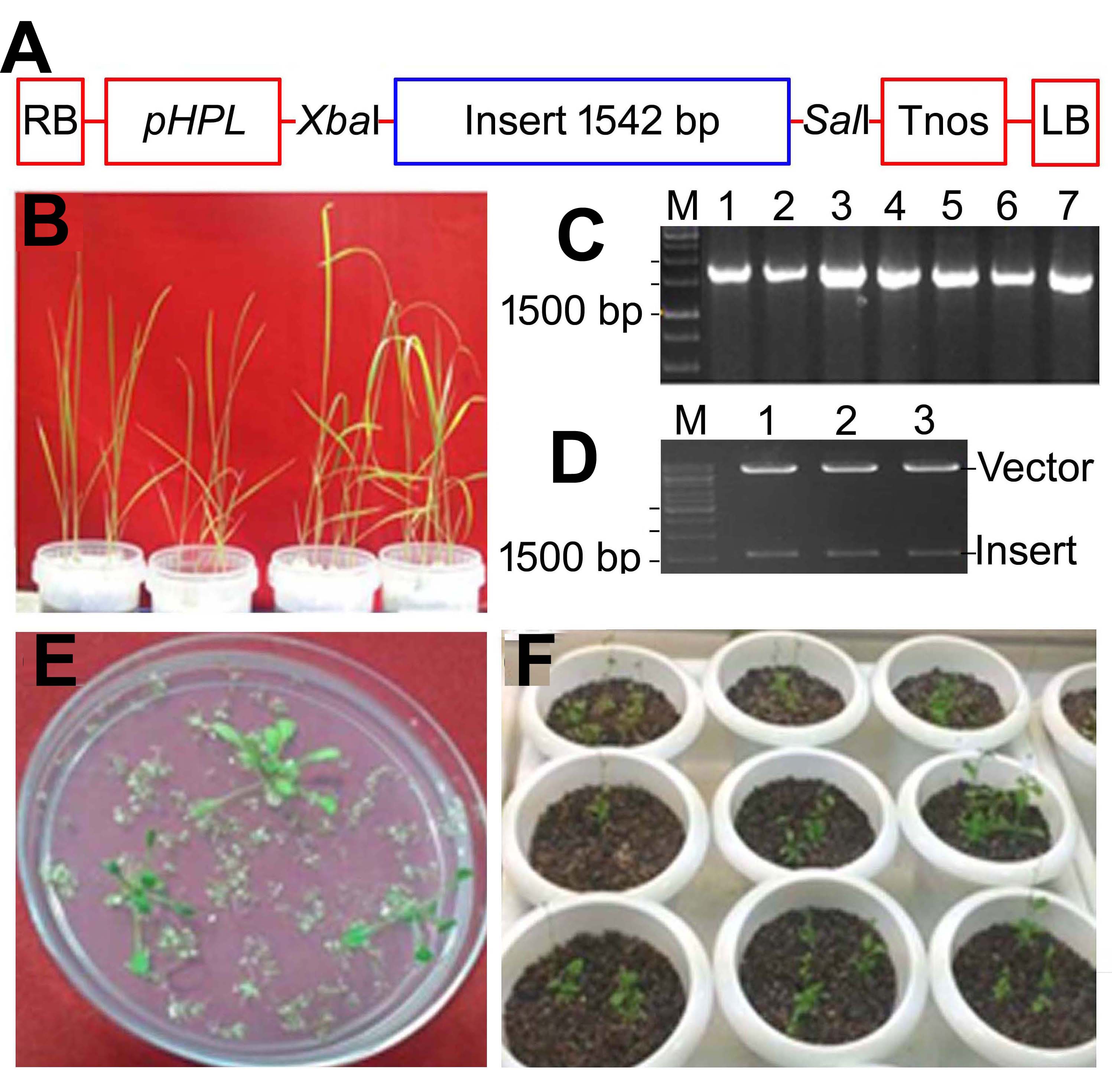

Supplement: Supplementary file 5 [file Image_4.JPEG]
